# MicroRNA‐495‐3p diminishes doxorubicin‐induced cardiotoxicity through activating AKT

**DOI:** 10.1111/jcmm.17230

**Published:** 2022-02-13

**Authors:** Jun Meng, Can Xu

**Affiliations:** ^1^ The First Affiliated Hospital Functional Department Hengyang Medical School University of South China Hengyang Hunan China; ^2^ The First Affiliated Hospital Department of Cardiology Hengyang Medical School University of South China Hengyang Hunan China

**Keywords:** AKT, doxorubicin‐induced cardiotoxicity, *miR‐495‐3p*, oxidative stress

## Abstract

Doxorubicin (Dox) is a broad‐spectrum antitumour agent; however, its clinical application is impeded due to the cumulative cardiotoxicity. The present study aims to investigate the role and underlying mechanisms of microRNA‐495‐3p (*miR*‐*495*‐*3p*) in Dox‐induced cardiotoxicity. Herein, we found that cardiac *miR*‐*495*‐*3p* expression was significantly decreased in Dox‐treated hearts, and that the *miR*‐*495*‐*3p* agomir could prevent oxidative stress, cell apoptosis, cardiac mass loss, fibrosis and cardiac dysfunction upon Dox stimulation. In contrast, the *miR*‐*495*‐*3p* antagomir dramatically aggravated Dox‐induced cardiotoxicity in mice. Besides, we found that the *miR*‐*495*‐*3p* agomir attenuated, while the *miR*‐*495*‐*3p* antagomir exacerbated Dox‐induced oxidative stress and cellular injury in vitro. Mechanistically, we demonstrated that *miR*‐*495*‐*3p* directly bound to the 3′‐untranslational region of phosphate and tension homology deleted on chromosome ten (PTEN), downregulated PTEN expression and subsequently activated protein kinase B (PKB/AKT) pathway, and that PTEN overexpression or AKT inhibition completely abolished the cardioprotective effects of the *miR*‐*495*‐*3p* agomir. Our study for the first time identify *miR*‐*495*‐*3p* as an endogenous protectant against Dox‐induced cardiotoxicity through activating AKT pathway in vivo and in vitro.

## 1 INTRODUCTION

Doxorubicin (Dox) is identified as a broad‐spectrum and efficient chemotherapeutic agent for multiple human solid and hematopoietic cancers; however, adverse cardiovascular events due to the cumulative cardiotoxicity extremely impede its clinical use.^1^, ^2^, ^3^ Despite the exact mechanisms of Dox‐induced cardiotoxicity remain unclear, reactive oxygen species (ROS) overproduction and oxidative damage are implicated in the pathogenesis of Dox‐induced cardiotoxicity.^4^, ^5^ Zhang et al.^6^ previously has revealed that Dox forms a ternary cleavage complex with topoisomerase‐II beta and DNA, which subsequently destroys the structure and function of mitochondria, and leads to excessive ROS generation. In addition, Dox treatment can increase the accumulation of iron inside the mitochondria, and then promote ROS amplification through a Fenton reaction.^7^ Moreover, the heart is especially vulnerable to oxidative damage due to the less active antioxidant defence and negligible regenerative capability.^8^ Accordingly, our recent study demonstrated that inhibiting oxidative stress significantly prevented Dox‐induced cardiotoxicity and dysfunction.^9^ Therefore, it is reasonable to treat Dox‐induced cardiotoxicity through inhibiting oxidative stress.

Protein kinase B (PKB/AKT) plays critical roles in cell survival and has been proposed as a promising therapeutic target of various cardiovascular diseases, including Dox‐induced cardiotoxicity.^10^, ^11^, ^12^ Findings from Zhang et al. showed that AKT activation significantly alleviated cardiomyocyte apoptosis and cardiac dysfunction in Dox‐treated mice. In addition, AKT activation can suppress the nuclear export and degradation of nuclear factor‐E2‐related factor 2 (NRF2), thereby protecting against Dox‐induced oxidative stress in the heart.^13^ Phosphate and tension homology deleted on chromosome ten (PTEN) is a major negative regulator of AKT phosphorylation and activation, and PTEN upregulation reduces AKT activation and amplifies oxidative damage in Dox‐treated hearts.^14^, ^15^ Consistently, Johnson et al.^16^ demonstrated that PTEN inhibitor significantly reduced apoptosis, cardiac remodelling and dysfunction in Dox‐treated mice. In contrast, overexpression of PTEN could exacerbate Dox‐induced cardiomyocyte apoptosis and oxidative stress through blocking AKT pathway.^17^ These findings identify AKT as a promising therapeutic candidate to treat Dox‐induced cardiotoxicity.

MicroRNAs (miRs) function as a class of endogenous negative gene regulators through binding to the 3′‐untranslational region (UTR) of target mRNAs. They are implicated in various biological processes, such as cell survival, death, senescence and canceration.^18^, ^19^, ^20^ Recent findings have indicated that miRNAs are also involved in regulating oxidative stress and Dox‐induced cardiotoxicity. Han et al.^21^ found that *miR*‐*330*‐*5p* contributed to Dox‐induced oxidative stress, DNA damage, cardiomyocyte injury and cardiac dysfunction. In addition, we also validated that *miR*‐*22* directly bound to the 3′‐UTR of silent information regulator 1 (SIRT1), and subsequently aggravated Dox‐induced oxidative stress, cardiomyocyte apoptosis and cardiac dysfunction, which were significantly attenuated by the *miR*‐*22* antagomir.^9^ *miR*‐*495*‐*3p* is well‐studied in human tumours, with high potency to inhibit tumour growth and chemoresistance.^22^, ^23^, ^24^ In addition, *miR*‐*495*‐*3p* is essential for regulating oxidative stress and cell survival. Lin et al.^25^ revealed that *miR*‐*495*‐*3p* upregulation promoted cell proliferation and inhibited apoptosis of tumour necrosis factor‐alpha‐induced human nucleus pulposus cells. Consistently, *miR*‐*495*‐*3p* elevation significantly suppressed oxidative stress, endothelial dysfunction and fibrosis in atherosclerotic mouse aortas.^26^ The present study aims to investigate the role and molecular mechanisms of *miR*‐*495*‐*3p* in Dox‐induced cardiotoxicity.

## 2 MATERIALS AND METHODS

### 2.1 Chemicals

Dox (#D1515), AKT inhibitor (#A6730) and ApopTag Plus In Situ Apoptosis Fluorescein Detection Kit (#S7111) were purchased from Sigma. Colorimetric Hydroxyproline Assay Kit (#ab222941), Colorimetric Lipid Peroxidation (MDA) Assay Kit (#ab118970), 3‐Nitrotyrosine ELISA Kit (#ab116691), 8‐hydroxy 2 deoxyguanosine ELISA Kit (8‐OHdG, #ab201734), Colorimetric Superoxide Dismutase (SOD) Activity Assay Kit (#ab65354), Colorimetric Catalase (CAT) Activity Assay Kit (#ab83464), Colorimetric NRF2 Transcription Factor Assay Kit (#ab207223), Nuclear Extraction Kit (#ab113474), Colorimetric Caspase‐3 Assay Kit (#ab39401) and LDH Assay Kit (Cytotoxicity, #ab65393) were purchased from Abcam. Mouse Troponin T, cardiac muscle (cTnT) ELISA Kit (CSB‐EL024016MO) and mouse L‐lactate dehydrogenase A chain ELISA Kit (CSB‐E17733m) were purchased from CUSABIO. 2,7‐dichlorodihydrofluorescein diacetate (DCFH‐DA, #D399) and Lipofectamine™ 3000 Transfection Reagent (#L3000015) were purchased from Thermo Fisher Scientific. Cell Counting Kit‐8 (CCK‐8, #C0037) was purchased from Beyotime Biotechnology. The agomir (#miR40003456‐4‐5), antagomir (#miR30003456‐4‐5) and respective controls (#miR4N0000001‐4‐5 for agomir control and #miR3N0000001‐4‐5 for antagomir control) of *miR*‐*495*‐*3p* were synthesized by RiboBio Co. Ltd. Adenovirus carrying rat PTEN or negative control (NC) and adeno‐associated virus serotype 9 (AAV9) carrying mouse PTEN or NC were generated by Hanbio Biotechnology Co., Ltd.

### 2.2 Experimental model of Dox‐induced cardiotoxicity

All experimental procedures were approved by our hospital and also in accordance with the Guidelines for the Care and Use of Laboratory Animals (NIH Publication, revised 1996). To generate Dox‐induced cardiotoxicity, male C57BL/6 mice (8–10‐week‐old) were randomly assigned to reduplicative intraperitoneal injections of Dox (4 mg/kg) weekly for 4 consecutive weeks according to previous studies by us and the others, whereas the control mice were treated with an equal volume of saline.^9^, ^27^ To overexpress or inhibit *miR*‐*495*‐*3p*, mice were intravenously treated with the agomir, antagomir or respective controls of *miR*‐*495*‐*3p* once 2 days for 10 times from the second day of Dox injection. Briefly, mice were fixed with the tails disinfected using 75% ethanol solution. Then, the agomir, antagomir or respective controls of *miR*‐*495*‐*3p* were injected from the tail vein, and special attention was paid to avoid liquid leakage. To inhibit AKT in vivo, mice were intraperitoneally injected with AKTi (20 mg/kg/day) for 14 days before sacrificed according to a previous study.^13^ The structure of AKTi was disclosed in the official website of Sigma https://www.sigmaaldrich.cn/CN/zh/product/sigma/a6730. To specifically overexpress PTEN in the myocardium, mice received a single injection of NC or PTEN carried by AAV9 from the tail vein at a concentration of 1 × 10^11^ viral genome per mouse at 4th week before Dox treatment.^28^ To observe the survival rate, mice were maintained for 6 weeks after the last Dox injection.

### 2.3 Determination of cardiac function

Cardiac function was performed by echocardiography and cardiac catheter according to previous studies.^9^, ^29^, ^30^ In brief, mice were anaesthetized with 2% isoflurane and then subjected to the echocardiographic assessment using a Vevo^®^ 2100 Imaging System (Visual Sonics) equipped with a 40 MHz MicroScan transducer (model MS‐550D). The functional parameters were calculated from at least five consecutive cardiac cycles. In addition, pressure‐volume loops were captured by a SPR‐839 microtip cardiac catheter (Millar Instruments), and analysed with the LabChart 7 software (ADInstruments) to evaluate haemodynamic parameters.

### 2.4 Western blot

Total proteins were extracted from the left ventricles of murine hearts or cultured cells using the RIPA lysis buffer as previously described.^31^, ^32^ Next, 20 μg total proteins were separated by 10% SDS‐PAGE, transferred onto PVDF membranes, blocked with 5% skim milk at room temperature and incubated with indicating primary antibodies at 4°C overnight. On the second day, the membranes were probed with horse radish peroxidase (HRP)‐conjugated secondary antibodies and visualized with an electrochemiluminescence reagent. The bands were analysed using an Image Lab software and normalized to GAPDH. The following antibodies were used at a 1:1000 dilution: anti‐NRF2 (#ab92946, Abcam), anti‐glyceraldehyde‐3‐phosphate dehydrogenase (GAPDH, #ab8245, Abcam), anti‐phosphorylated AKT (p‐AKT, #4060, Cell Signaling Technology), anti‐total AKT (t‐AKT, #4685, Cell Signaling Technology) and anti‐PTEN (#ab267787, Abcam).

### 2.5 Quantitative real‐time PCR

Total RNA was extracted using Trizol reagent, and reverse transcription was performed with the Prime Script RT Master Mix (Takara) and random or oligo (dT) primer. Then, quantitative real‐time PCR was conducted using a SYBR^®^ Premix Ex Taq^TM^ kit (Takara) with GAPDH and U6 used as internal controls for mRNA and miRNA respectively.^31^, ^33^, ^34^ The primer sequences were listed as follows: collagen I, forward, 5′‐AGGCTTCAGTGGTTTGGATG‐3′ and reverse, 5′‐CACCAACAGCACCATCGTTA‐3′; collagen III, forward, 5′‐CCCAACCCAGAGATCCCATT‐3′ and reverse, 5′‐GAAGCA CAGGAGCAGGTGTAGA‐3′; B cell leukaemia/lymphoma‐2 (BCL‐2), forward, 5′‐GTCGCTACCGTCGTGACTTC‐3′ and reverse, 5′‐CAGACATGCACCTACCCAGC‐3′; BCL‐2‐associated X protein (BAX), forward, 5′‐TGAAGACAGGGGCCTTTTTG‐3′ and reverse, 5′‐AATTCGCCGGAGACACTCG‐3′; GAPDH, forward, 5′‐CGTGCCGCCTGGAGAAACC‐3′ and reverse, 5′‐TGGAAGAG TGGGAGTTGCTGTTG‐3′.

### 2.6 Collagen content measurements

Total collagen content in the left ventricles was determined by measuring the level of hydroxyproline, a major component of collagen, as previously described.^35^ Briefly, left ventricles were homogenized and hydrolysed in NaOH at 120°C for 1 h, and then neutralized by concentrated HCl. The lysates were then centrifuged at 10,000 *g* for 5 min to remove the insoluble debris, and then the supernatants were incubated with chloramine T and oxidation buffer according to the manufacturer’s instructions at room temperature for 20 min. Then, the mixture was reacted with Developer at 37°C for 5 min, and DMAB concentrate solution 65°C for 45 min, and the absorbance was measured at 560 nm.

### 2.7 Analysis of serum cardiac injury biomarkers

Serum samples were collected from mice at 3 days after the last Dox injection and then were used to detect the levels of cTnT and LDH to determine cardiac injury according to the manufacturer’s instructions.

### 2.8 Oxidative stress detection

Reactive oxygen species generation was detected using a DCFH‐DA method as previously described.^36^, ^37^ Briefly, fresh left ventricles or cells were homogenized and incubated with DCFH‐DA (20 μmol/L) at 37°C for 3 h, and the fluorescence was detected at an excitation wavelength of 488 nm and emission wavelength of 525 nm. The levels of MDA, 3‐NT and 8‐OHdG were detected using commercial kits to evaluate the peroxidation of lipid, protein and nucleic acid. Total SOD and CAT activities were measured to determine endogenous antioxidant capacities according to the manufacturer’s instructions. Nuclear extracts from fresh left ventricles or cells were prepared using a nuclear extraction kit according to the manufacturer’s instructions, and then incubated in plates pre‐coated with oligonucleotide containing NRF2 consensus binding site, which was then reacted with anti‐NRF2 antibody and HRP‐conjugated secondary antibody. Finally, the absorbance was detected at 450 nm with a reference wavelength of 665 nm.

### 2.9 Cell apoptosis quantification

TdT‐mediated dUTP Nick End Labelling (TUNEL)+ cells were detected by a commercial kit as we previously described, and the percent of TUNEL+nuclei was calculated as the apoptotic index.^9^ In addition, caspase‐3 activity was determined to evaluate cell apoptosis according to our previous study.^9^ Briefly, fresh cell lysates were prepared and centrifuged at 10,000 *g* for 1 min to obtain the cell‐free supernatants, which were then incubated with DVED‐pNA (200 μmol/L) at 37°C for 2 h and detected at 400 nm.

### 2.10 Cell culture and treatments

H9C2 cells were purchased from ATCC and cultured in DMEM containing 10% FBS as we previously described.^9^ To overexpress or inhibit *miR*‐*495*‐*3p*, cells were transfected with the agomir, antagomir and respective controls of *miR*‐*495*‐*3p* at a concentration of 50 nmol/L using Lipofectamine™ 3000 Transfection Reagent for 24 h, and then stimulated with or without Dox (1 μmol/L) for an additional 24 h.^38^ To inhibit AKT, cells were treated with AKTi (1 μmol/L) for 30 min.^13^ To overexpress PTEN, cells were infected with adenovirus‐carried PTEN at a multiple of infection of 30.

### 2.11 Cell viability and injury

Cell viability was determined by the CCK‐8 method as previously described.^39^, ^40^ In brief, stimulated cells were incubated with the CCK‐8 solution at 37°C for 30 min, and then the absorbance was detected at 450 nm with a reference wavelength of 650 nm. LDH releases to the medium were detected using a commercial kit according to the manufacturer's instructions to evaluate cell injury.

### 2.12 Luciferase reporter assay

Wild type (WT) or mutant (MUT) PTEN 3′‐UTR were amplified from the genomic DNA and then cloned into the psi‐CHECK2 luciferase reporter plasmid (Promega), which were then co‐transfected with the *miR*‐*495*‐*3p* agomir or control to HEK293T cells. 48 h after transfection, cells were lysed, and the luciferase activity was determined by a dual luciferase reporter system (Promega).^9^, ^41^, ^42^

### 2.13 Statistical analysis

All results were expressed as the mean ± standard deviation and analysed by SPSS 23.0 software. Two‐tailed Student's *t*‐test was conducted to compare differences between two groups, while comparisons among three or more groups were performed by one‐way ANOVA, followed by Tukey post hoc test. Survival rate was evaluated by the Kaplan–Meier method and survival curves were compared using the Mantel–Cox log‐rank test. *p* < 0.05 was considered statistically significant.

## 3 RESULTS

### 3.1 *miR*‐*495*‐*3p* agomir alleviates Dox‐induced cardiotoxicity in mice

**FIGURE 1:**
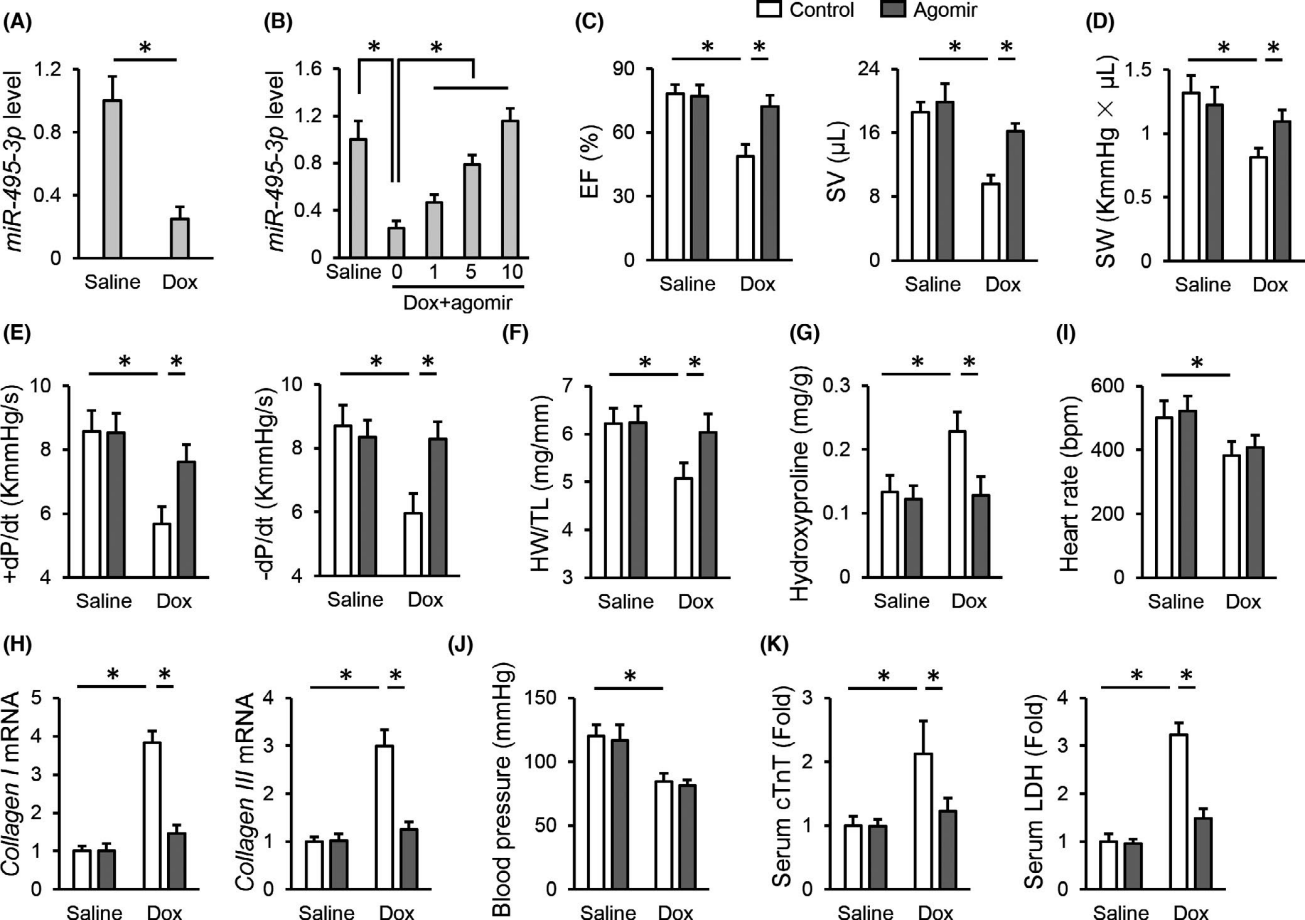
*miR*‐*495*‐*3p* agomir alleviates Dox‐induced cardiotoxicity in mice. (A) *miR*‐*495*‐*3p* expression in the heart with or without Dox treatment. (B) *miR*‐*495*‐*3p* expression in Dox‐treated hearts in the presence or absence of the *miR*‐*495*‐*3p* agomir. (C‐E) Cardiac function as determined by the EF, SV, SW and ±dP/dt in mice treated with or without the *miR*‐*495*‐*3p* agomir. (F) Quantitative results of HW/TL. (G) Cardiac fibrosis as determined by the level of hydroxyproline in the heart. (H) The mRNA levels of *collagen I* and *collagen III* in the heart. (I,J) Quantitative results of heart rate and blood pressure. (K) Relative levels of serum cTnT and LDH in mice treated with or without the *miR*‐*495*‐*3p* agomir. *N* = 6 per group. All results were expressed as the mean ± standard deviation and *p* < 0.05 was considered statistically significant

We first examined cardiac *miR*‐*495*‐*3p* expression upon Dox stimulation, and found that *miR*‐*495*‐*3p* level was significantly decreased in Dox‐treated hearts (Figure 1A). To investigate the role of *miR*‐*495*‐*3p*, mice were injected with the *miR*‐*495*‐*3p* agomir through tail vein. As shown in Figure 1B, injections with the *miR*‐*495*‐*3p* agomir at a dose of 10 nmol/g/day completely restored cardiac *miR*‐*495*‐*3p* expression upon Dox stimulation; therefore, we used this dose for further study. As expected, Dox injection caused cardiac dysfunction in mice, as evidenced by the decreased ejection fraction (EF), stroke volume (SV), stroke work (SW) and ±dP/dt, which were significantly alleviated by the *miR*‐*495*‐*3p* agomir (Figure 1C–E). In addition, treatment with the *miR*‐*495*‐*3p* agomir also prevented the loss of cardiac mass in Dox‐treated mice, as evidenced by the increased heart weight (HW)/tibial length (TL) (Figure 1F). Fibrosis is a key feature of Dox‐induced cardiotoxicity and increases cardiac stiffness.^43^ Intriguingly, the *miR*‐*495*‐*3p* agomir significantly suppressed cardiac fibrosis upon Dox stimulation (Figure 1G,H). Yet, no alteration of heart rate or blood pressure was found in the *miR*‐*495*‐*3p* agomir‐treated mice with or without Dox injection (Figure 1I,J). Meanwhile, the elevated levels of serum cTnT and LDH were also reduced by the *miR*‐*495*‐*3p* agomir in Dox‐treated mice (Figure 1K). Moreover, Dox‐caused mortality in mice was largely prevented with the *miR*‐*495*‐*3p* agomir (55% in Dox+miR‐*495*‐*3p* agomir control vs. 85% in Dox+miR‐*495*‐*3p* agomir). Taken together, our data reveal that the *miR*‐*495*‐*3p* agomir alleviates Dox‐induced cardiotoxicity in mice.

### 3.2 *miR*‐*495*‐*3p* antagomir aggravates Dox‐induced cardiotoxicity in mice

**FIGURE 2:**
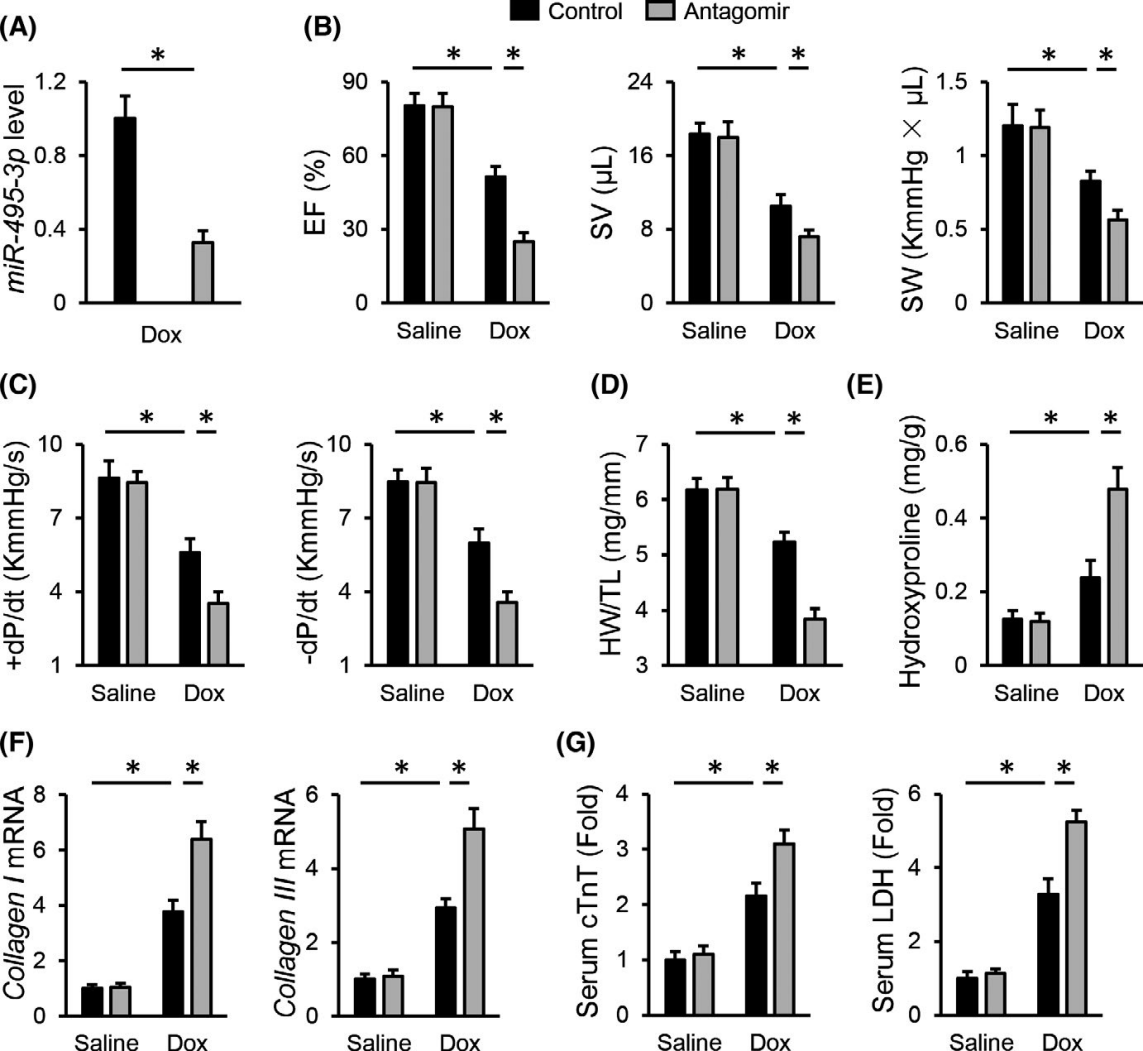
*miR*‐*495*‐*3p* antagomir aggravates Dox‐induced cardiotoxicity in mice. (A) *miR*‐*495*‐*3p* expression in Dox‐treated hearts in the presence or absence of the *miR*‐*495*‐*3p* antagomir. (B,C) Cardiac function as determined by the EF, SV, SW and ±dP/dt in mice treated with or without the *miR*‐*495*‐*3p* antagomir. (D) Quantitative results of HW/TL. (E) Cardiac fibrosis as determined by the level of hydroxyproline in the heart. (F) The mRNA levels of *collagen I* and *collagen III* in the heart. (G) Relative levels of serum cTnT and LDH in mice treated with or without the *miR*‐*495*‐*3p* antagomir. *N* = 6 per group. All results were expressed as the mean ± standard deviation and *p* < 0.05 was considered statistically significant

Next, we investigated whether inhibiting *miR*‐*495*‐*3p* with the *miR*‐*495*‐*3p* antagomir would aggravate Dox‐induced cardiotoxicity in mice, and the efficiency was presented in Figure 2A. As shown in Figure 2B,C, mice treated with the *miR*‐*495*‐*3p* antagomir displayed further compromised cardiac function upon Dox injection, as evidenced by the decreased EF, SV, SW and ±dP/dt. As expected, Dox‐induced cardiac mass loss and fibrotic remodelling were further aggravated by the *miR*‐*495*‐*3p* antagomir (Figure 2D–F). In addition, treatment with the *miR*‐*495*‐*3p* antagomir dramatically increased serum cTnT and LDH levels in mice upon Dox injection (Figure 2G). More importantly, we observed that the *miR*‐*495*‐*3p* antagomir‐treated mice all died within 5 weeks after the last Dox injection, indicating a higher mortality rate caused by the *miR*‐*495*‐*3p* antagomir. Taken together, we determine that the *miR*‐*495*‐*3p* antagomir aggravates Dox‐induced cardiotoxicity in mice.

### 3.3 *miR*‐*495*‐*3p* agomir inhibits oxidative damage and apoptosis in Dox‐treated hearts

**FIGURE 3:**
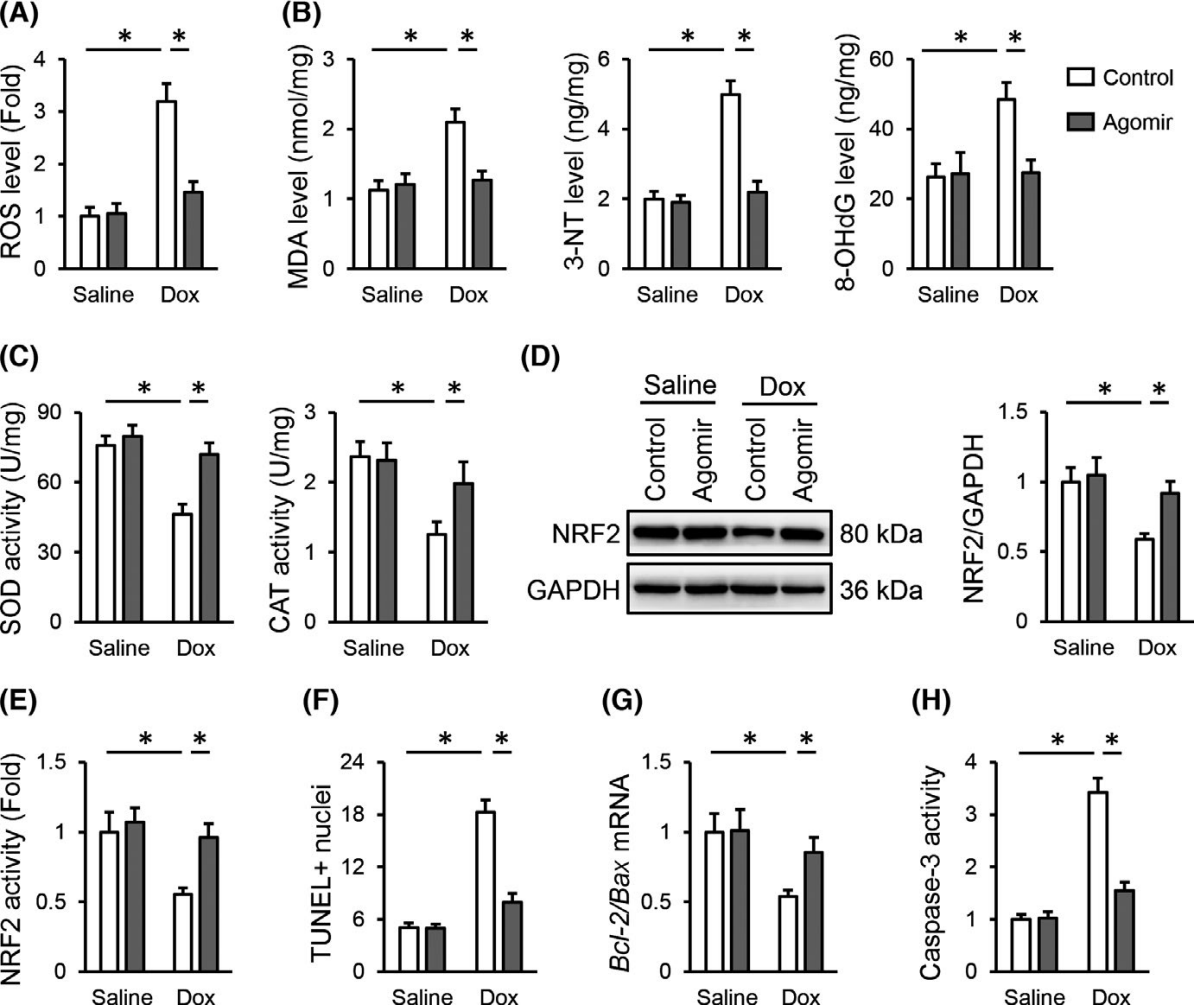
*miR*‐*495*‐*3p* agomir inhibits oxidative damage and apoptosis in Dox‐treated hearts. (A) ROS generation in the *miR*‐*495*‐*3p* agomir‐treated hearts upon Dox injection. (B) The levels of MDA, 3‐NT and 8‐OHdG in the heart. (C) Quantitative results of total SOD and CAT activities. (D) Representative images of NRF2 protein and quantitative data. (E) Nuclear extracts from fresh left ventricles or cells were prepared using a Nuclear Extraction Kit according to the manufacturer's instructions, and then incubated in plates pre‐coated with oligonucleotide containing NRF2 consensus binding site, which was then reacted with anti‐NRF2 antibody and HRP‐conjugated secondary antibody. Finally, the absorbance was detected at 450 nm with a reference wavelength of 665 nm to detect NRF2 transcription activity. (F) Cell apoptosis as determined by the quantification of TUNEL‐positive nuclei. (G) Ratio of *Bcl*‐*2* to *Bax* mRNA levels. (H) Quantitative results of caspase‐3 activity in the heart. *N* = 6 per group. All results were expressed as the mean ± standard deviation and *p* < 0.05 was considered statistically significant

Oxidative stress plays critical roles in the initiation and progression of Dox‐induced cardiotoxicity.^2^, ^29^ Accordingly, we detected a significant increase of ROS accumulation in the heart with Dox treatment, which was reduced by injections of the *miR*‐*495*‐*3p* agomir (Figure 3A). ROS overproduction results in the peroxidation of lipid, protein and nucleic acid, eventually leading to cell apoptosis and cardiac dysfunction. In line with the lower ROS levels, the *miR*‐*495*‐*3p* agomir also decreased the generations of MDA, 3‐NT and 8‐OHdG in Dox‐stimulated hearts (Figure 3B). SOD and CAT are endogenous antioxidant enzymes that help to scavenge excessive free radicals. In line with previous studies by us and the others, Dox injection dramatically suppressed the activities of total SOD and CAT, which, however, were partially restored by the *miR*‐*495*‐*3p* agomir (Figure 3C).^3^, ^9^ NRF2 is a central transcription factor to regulate the expression of various antioxidant enzymes, and NRF2 downregulation exacerbates Dox‐induced cardiotoxicity.^13^, ^44^ Intriguingly, Dox‐induced inhibition of NRF2 expression and activity was significantly enhanced by the *miR*‐*495*‐*3p* agomir (Figure 3D,E). Meanwhile, we detected fewer apoptotic cells in the *miR*‐*495*‐*3p* agomir‐treated hearts upon Dox stimulation, accompanied with the increased *Bcl*‐*2*/*Bax* mRNA level and decreased caspase‐3 activity (Figure 3F–H). Taken together, our findings indicate that the *miR*‐*495*‐*3p* agomir inhibits oxidative damage and apoptosis in Dox‐treated hearts.

### 3.4 *miR*‐*495*‐*3p* antagomir exacerbates oxidative damage and apoptosis in Dox‐treated hearts

**FIGURE 4:**
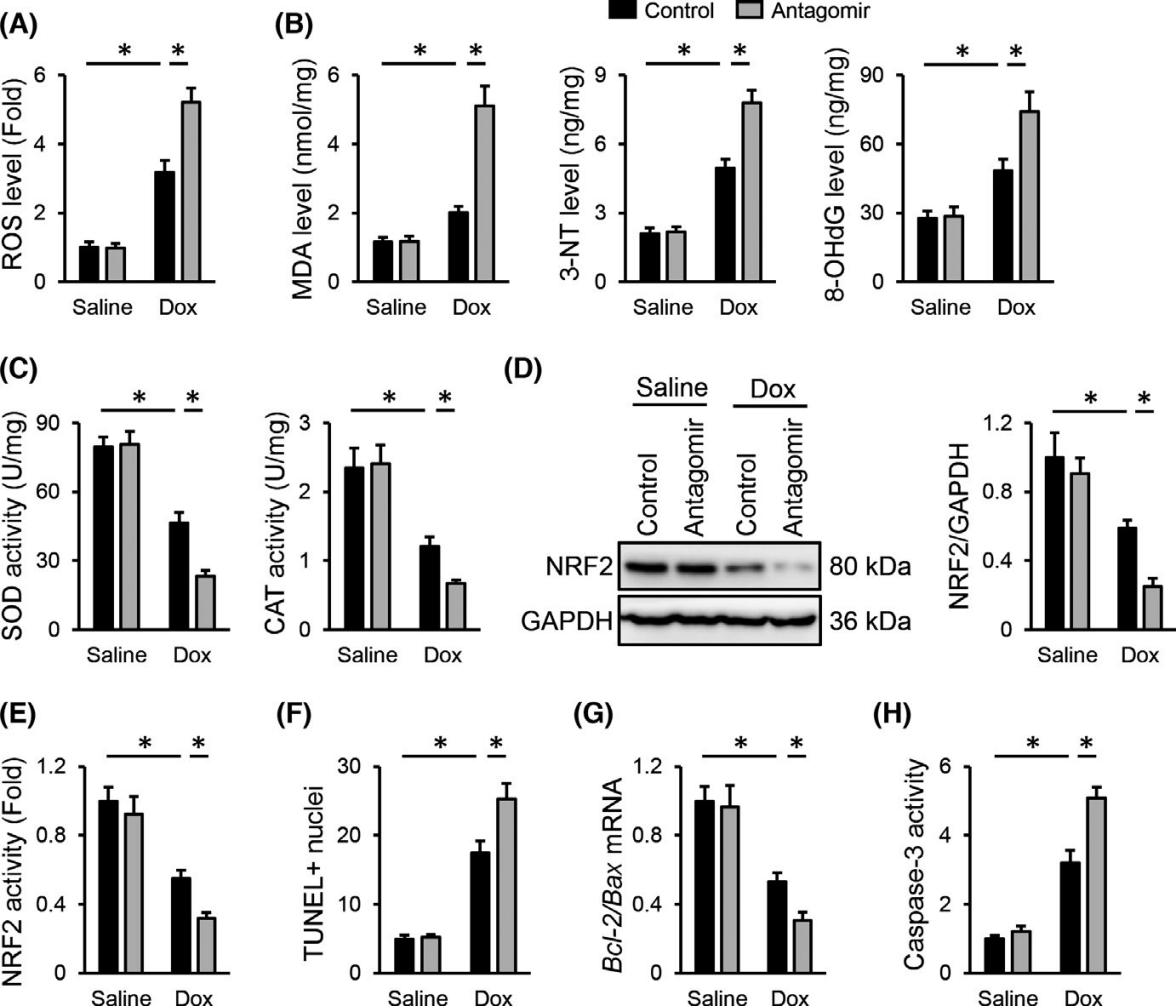
*miR*‐*495*‐*3p* antagomir exacerbates oxidative damage and apoptosis in Dox‐treated hearts. (A) ROS generation in the *miR*‐*495*‐*3p* antagomir‐treated hearts upon Dox injection. (B) The levels of MDA, 3‐NT and 8‐OHdG in the heart. (C) Quantitative results of total SOD and CAT activities. (D) Representative images of NRF2 protein and quantitative data. (E) Quantitative results of NRF2 transcription activity. (F) Cell apoptosis as determined by the quantification of TUNEL‐positive nuclei. (G) Ratio of *Bcl*‐*2* to *Bax* mRNA levels. (H) Quantitative results of caspase‐3 activity in the heart. *N* = 6 per group. All results were expressed as the mean ± standard deviation and *p* < 0.05 was considered statistically significant

In contrast with the antioxidant role of the *miR*‐*495*‐*3p* agomir, Dox‐induced ROS generation was further amplified by the *miR*‐*495*‐*3p* antagomir, as evidenced by the increased ROS, MDA, 3‐NT and 8‐OHdG levels (Figure 4A,B). Accordingly, treatment with the *miR*‐*495*‐*3p* antagomir also suppressed the activities of total SOD and CAT upon Dox injection (Figure 4C). Meanwhile, mice treated with the *miR*‐*495*‐*3p* antagomir displayed lower NRF2 expression and activity than those treated with the antagomir control upon Dox injection (Figure 4D,E). In addition, Dox‐induced apoptosis of cardiac cells was also aggravated by the *miR*‐*495*‐*3p* antagomir, as evidenced by the increased TUNEL+nuclei, caspase‐3 activity and decreased *Bcl*‐*2*/*Bax* mRNA level (Figure 4F–H). Collectively, we demonstrate that the *miR*‐*495*‐*3p* antagomir exacerbates oxidative damage and apoptosis in Dox‐treated hearts.

### 3.5 *miR*‐*495*‐*3p* modulates Dox‐induced oxidative stress and cellular injury in vitro

**FIGURE 5:**
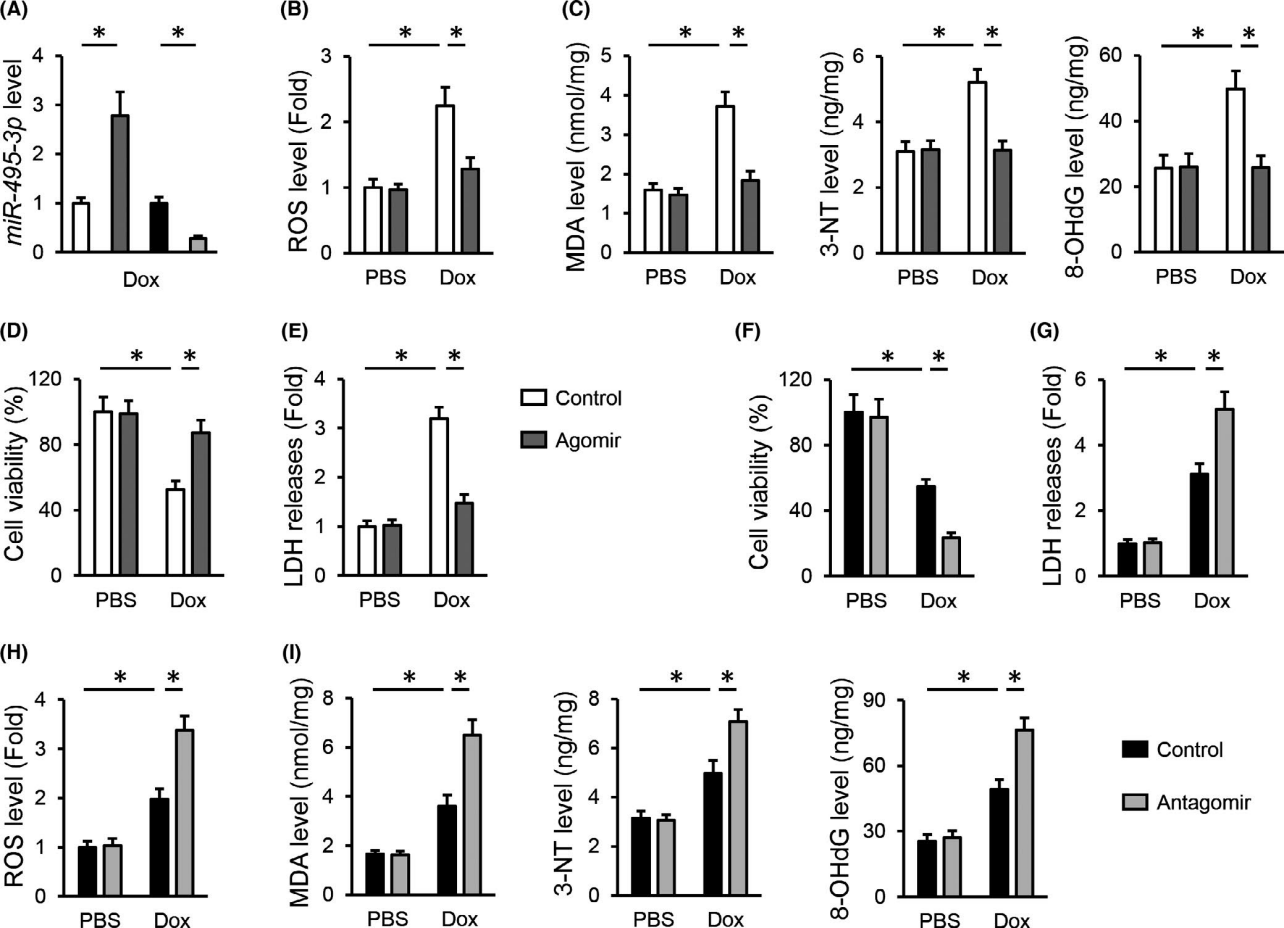
*miR*‐*495*‐*3p* modulates Dox‐induced oxidative stress and cellular injury in vitro. (A) *miR*‐*495*‐*3p* expression in H9C2 cells treated with the agomir or antagomir of *miR*‐*495*‐*3p* upon Dox stimulation. (B) ROS generation in the *miR*‐*495*‐*3p* agomir‐treated H9C2 cells upon Dox stimulation. (C) The levels of MDA, 3‐NT and 8‐OHdG in the *miR*‐*495*‐*3p* agomir‐treated H9C2 cells. (D,E) Quantitative results of cell viability and LDH releases to the medium in H9C2 cells treated with the *miR*‐*495*‐*3p* agomir. (F,G) Quantitative results of cell viability and LDH releases to the medium in H9C2 cells treated with the *miR*‐*495*‐*3p* antagomir. (H) ROS generation in the *miR*‐*495*‐*3p* antagomir‐treated H9C2 cells upon Dox stimulation. (I) The levels of MDA, 3‐NT and 8‐OHdG in the *miR*‐*495*‐*3p* antagomir‐treated H9C2 cells. *N* = 6 per group. All results were expressed as the mean ± standard deviation and *p* < 0.05 was considered statistically significant

Then, we evaluated the role of *miR*‐*495*‐*3p* in Dox‐treated H9C2 cells in vitro, and the efficiency was presented in Figure 5A. Consistent with the in vivo data, Dox‐induced ROS generation in H9C2 cells was significantly attenuated by the *miR*‐*495*‐*3p* agomir, accompanied by a decreased peroxidation of lipid, protein and nucleic acid (Figure 5B,C). In addition, the *miR*‐*495*‐*3p* agomir also prevented Dox‐induced cell death and injury, as evidenced by the increased cell viability and decreased LDH releases (Figure 5D,E). In contrast, the decreased survival rate and increased LDH releases in Dox‐treated H9C2 cells were further amplified by the *miR*‐*495*‐*3p* antagomir (Figure 5F,G). In addition, the *miR*‐*495*‐*3p* antagomir‐treated cells also exhibited higher ROS generation and severe oxidative damage (Figure 5H,I). Overall, we validate that the *miR*‐*495*‐*3p* agomir attenuates, while the *miR*‐*495*‐*3p* antagomir aggravates Dox‐induced oxidative stress and cellular injury in vitro.

### 3.6 *miR*‐*495*‐*3p* agomir prevents Dox‐induced cardiotoxicity through activating AKT in vivo and in vitro

**FIGURE 6:**
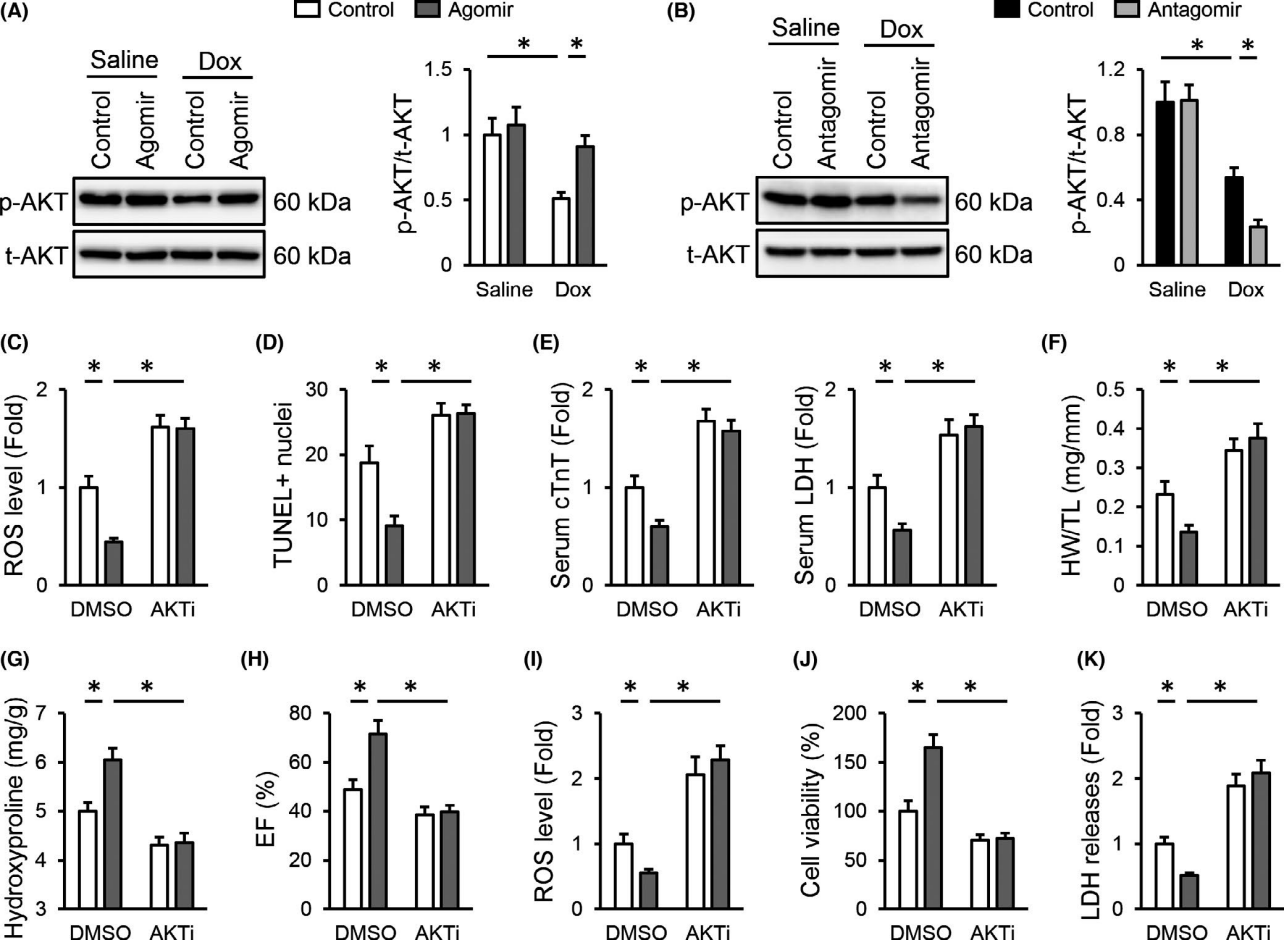
*miR*‐*495*‐*3p* agomir prevents Dox‐induced cardiotoxicity through activating AKT in vivo and in vitro. (A,B) Representative Western blot images and quantitative data. (C) ROS generation in the *miR*‐*495*‐*3p* agomir‐treated hearts with or without AKTi injection upon Dox stimulation. (D) Cell apoptosis as determined by the quantification of TUNEL‐positive nuclei. (E) Relative levels of serum cTnT and LDH in mice treated with or without the *miR*‐*495*‐*3p* agomir in the presence or absence of AKTi. (F) Quantitative results of HW/TL. (G) Cardiac fibrosis as determined by the level of hydroxyproline in the heart. (H) EF in the *miR*‐*495*‐*3p* agomir‐treated mice with or without AKTi injection upon Dox stimulation. (I) ROS generation in the *miR*‐*495*‐*3p* agomir‐treated H9C2 cells upon Dox stimulation in the presence or absence of AKTi. (J,K) Quantitative results of cell viability and LDH releases to the medium in Dox‐stimulated H9C2 cells treated with the *miR*‐*495*‐*3p* agomir in the presence or absence of AKTi. *N* = 6 per group. All results were expressed as the mean ± standard deviation and *p* < 0.05 was considered statistically significant

Next, we explored the underlying mechanisms that mediate the cardioprotective effects of the *miR*‐*495*‐*3p* agomir in vivo and in vitro. AKT plays critical roles in cell survival and oxidative stress, and activating AKT is sufficient to alleviate Dox‐induced cardiotoxicity.^13^ Interestingly, we found that Dox‐induced suppression on AKT phosphorylation was prevented by the *miR*‐*495*‐*3p* agomir, but further aggravated by the *miR*‐*495*‐*3p* antagomir (Figure 6A,B). To determine whether the *miR*‐*495*‐*3p* agomir prevented Dox‐induced cardiotoxicity through activating AKT, mice were treated with AKTi to inhibit AKT activity as previously described. As shown in Figure 6C,D, AKT inhibition completely abolished the antioxidant and antiapoptotic effects of the *miR*‐*495*‐*3p* agomir. In addition, the decreased serum cTnT and LDH levels in the *miR*‐*495*‐*3p* agomir‐treated mice upon Dox injection were significantly increased in those treated with AKTi (Figure 6E). Meanwhile, the alleviation of cardiac mass loss and fibrosis by the *miR*‐*495*‐*3p* agomir was also abrogated in the presence of AKTi (Figure 6F,G). Consistent with the molecular alterations, the *miR*‐*495*‐*3p* agomir failed to improve Dox‐induced cardiac dysfunction in AKTi‐treated mice upon Dox injection (Figure 6H). In addition, we also evaluated the necessity of AKT in mediating the beneficial effects of the *miR*‐*495*‐*3p* agomir in vitro. As shown in Figure 6I–K, the protective effects of the *miR*‐*495*‐*3p* agomir against Dox‐induced oxidative stress and cellular injury were significantly blocked by AKTi. Collectively, our observations indicate that the *miR*‐*495*‐*3p* agomir prevents Dox‐induced cardiotoxicity through activating AKT in vivo and in vitro.

### 3.7 *miR*‐*495*‐*3p* agomir activates AKT through downregulating PTEN

**FIGURE 7:**
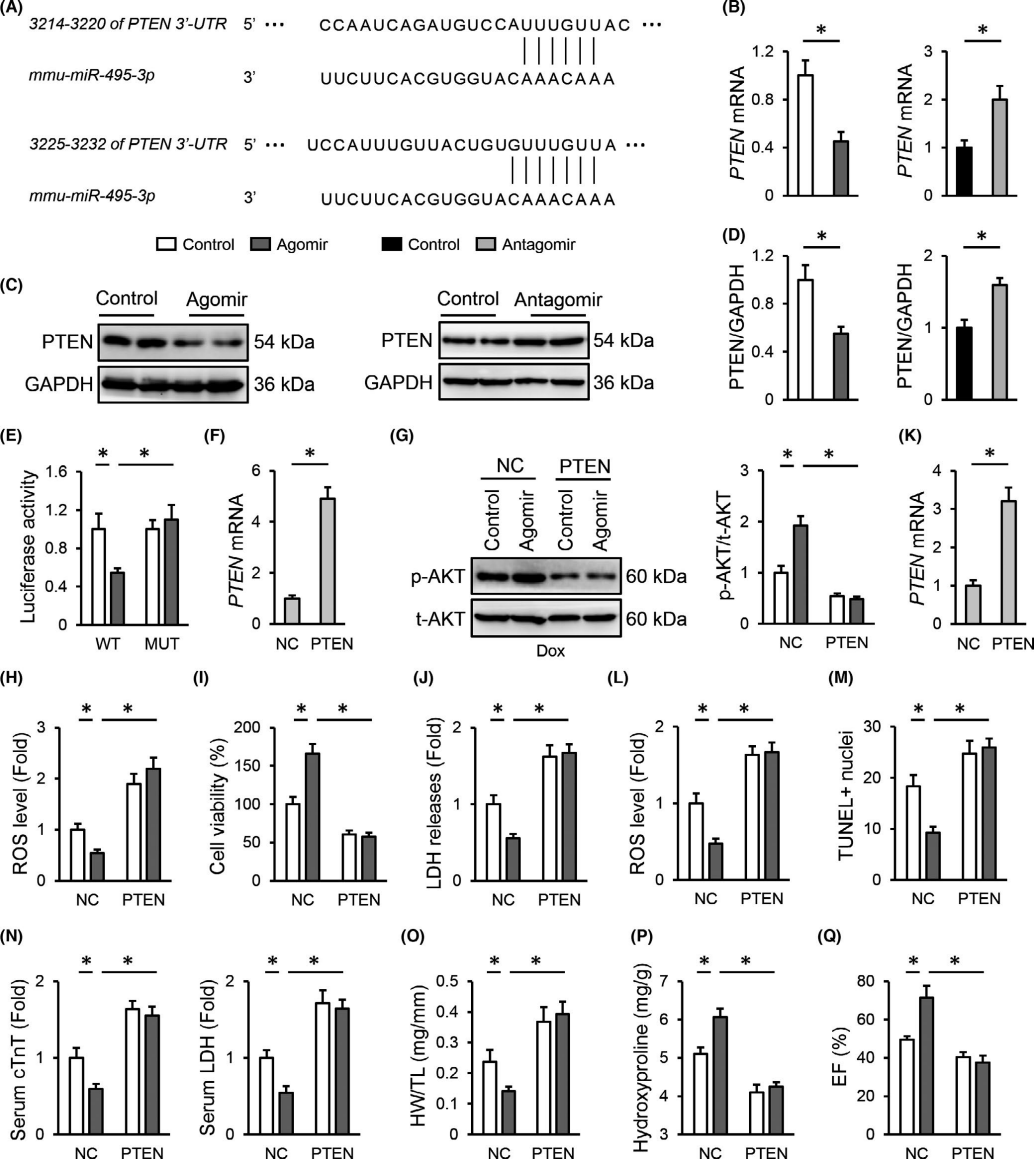
*miR*‐*495*‐*3p* agomir activates AKT through downregulating PTEN. (A) Putative binding sites of *PTEN* 3′‐UTR and *miR*‐*495*‐*3p* were predicted by a bioinformatic software TargetScan. (B–D) Relative mRNA and protein levels of PTEN in H9C2 cells treated with the *miR*‐*495*‐*3p* agomir or antagomir. (E) Quantitative results of luciferase activity. (F) *PTEN* expression in H9C2 cells infected with the adenovirus carrying PTEN or NC. (G) AKT phosphorylation in the *miR*‐*495*‐*3p* agomir‐treated H9C2 cells upon Dox stimulation with or without PTEN overexpression. (H) ROS generation in the *miR*‐*495*‐*3p* agomir‐treated H9C2 cells upon Dox stimulation with or without PTEN overexpression. (I,J) Quantitative results of cell viability and LDH releases to the medium. (K) *PTEN* expression in the heart infected with the AAV9 vectors carrying PTEN or NC. (L) ROS generation in the *miR*‐*495*‐*3p* agomir‐treated hearts with or without PTEN overexpression upon Dox stimulation. (M) Cell apoptosis as determined by the quantification of TUNEL‐positive nuclei. (N) Relative levels of serum cTnT and LDH in PTEN‐overexpressed mice treated with or without the *miR*‐*495*‐*3p* agomir. (O) Quantitative results of HW/TL. (P) Cardiac fibrosis as determined by the level of hydroxyproline in the heart. (Q) EF in the *miR*‐*495*‐*3p* agomir‐treated mice with or without PTEN overexpression upon Dox stimulation. *N* = 6 per group. All results were expressed as the mean ± standard deviation and *p* < 0.05 was considered statistically significant

Finally, we tried to unveil the molecular basis through which the *miR*‐*495*‐*3p* agomir activated AKT. Using the TargetScan software, PTEN, a classic negative regulator of AKT phosphorylation and activation was identified, with two putative conserved binding sites on PTEN 3′‐UTR of *miR*‐*495*‐*3p* (Figure 7A). Meanwhile, we found that the *miR*‐*495*‐*3p* agomir reduced, while the *miR*‐*495*‐*3p* antagomir elevated PTEN mRNA and protein levels in H9C2 cells (Figure 7B–D). Luciferase reporter assay further validated the direct interaction between *miR*‐*495*‐*3p* and PTEN 3′‐UTR (Figure 7E). To determine the necessity of PTEN in the *miR*‐*495*‐*3p* agomir‐mediated AKT activation and cardioprotection, H9C2 cells were infected with adenovirus to overexpress PTEN in vitro (Figure 7F). As shown in Figure 7G, PTEN overexpression completely abolished the *miR*‐*495*‐*3p* agomir‐induced AKT activation upon Dox treatment in H9C2 cells. Accordingly, the decreased oxidative stress and cellular injury in the *miR*‐*495*‐*3p* agomir‐treated cells were also prevented in those with PTEN overexpression, as determined by the increased ROS generation, LDH releases and decreased cell viability (Figure 7H–J). In addition, mice were also injected with AAV9 vectors carrying either PTEN or NC to specifically overexpress PTEN in the myocardium (Figure 7K). As shown in Figure 7L,M, the *miR*‐*495*‐*3p* agomir significantly reduced Dox‐induced oxidative stress and cell apoptosis in the heart, but failed to do so in those with PTEN overexpression. Accordingly, the *miR*‐*495*‐*3p* agomir‐induced improvement of cardiac injury, cardiac mass loss and fibrotic remodelling was also blocked in PTEN‐overexpressed mice (Figure 7N–P). In addition, PTEN overexpression significantly abrogated functional restoration in the *miR*‐*495*‐*3p* agomir‐treated mice upon Dox injection (Figure 7Q). Overall, our findings determine that the *miR*‐*495*‐*3p* agomir activates AKT through downregulating PTEN.

## 4 DISCUSSION

**FIGURE 8:**
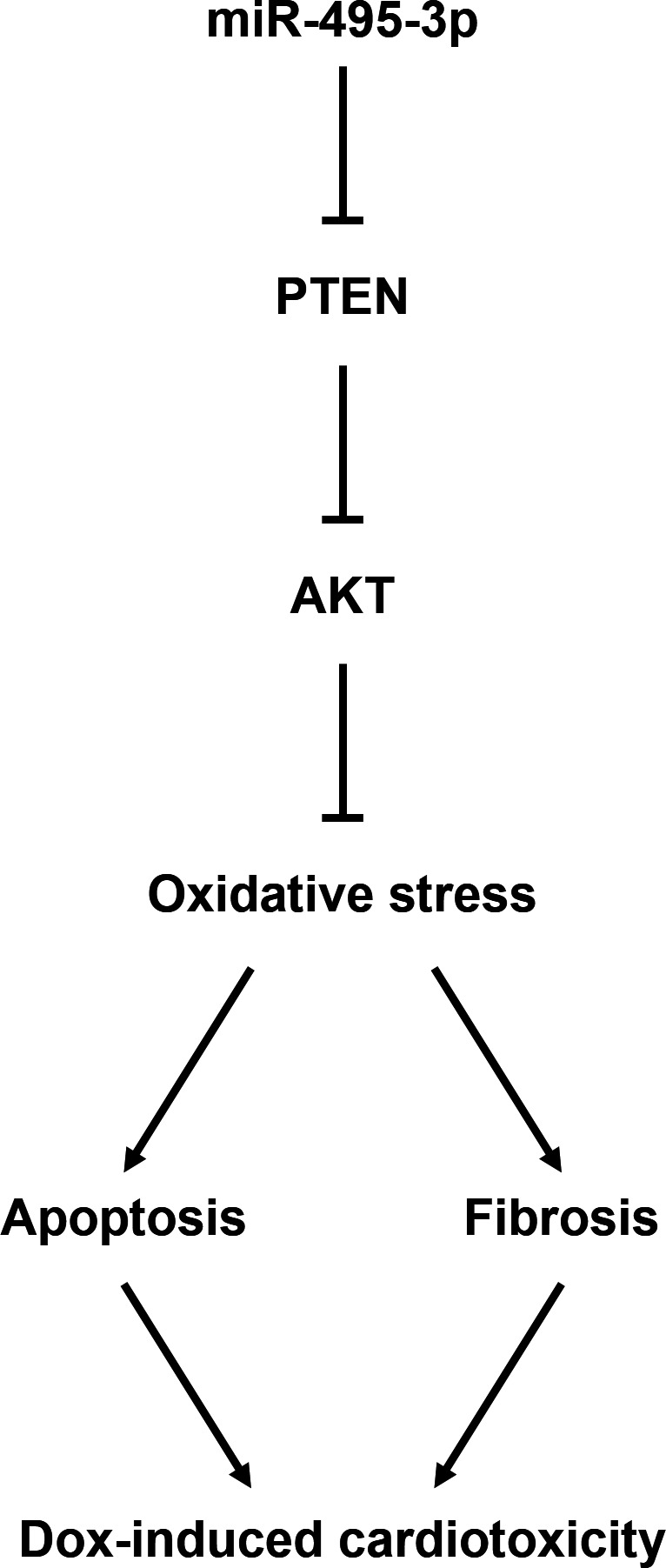
Working model of *miR*‐*495*‐*3p* in Dox‐induced cardiotoxicity. *miR*‐*495*‐*3p* directly binds to the 3′‐UTR of PTEN, downregulates PTEN expression and subsequently activates AKT pathway, thereby preventing Dox‐induced oxidative damage and cardiac dysfunction

Chemotherapy is one of the major approaches to treat human cancers, and Dox, belonging to the family of anthracyclines antibiotics, has attracted extensive interests due to its broad‐spectrum antitumour capacities. Unfortunately, the clinical application of Dox is extremely impeded because of its cumulative cardiotoxicity.^45^, ^46^ Dexrazoxane is the only FDA‐approved cardioprotectant to treat Dox‐induced cardiotoxicity; however, long‐term use of high doses of dexrazoxane can lead to severe hepatotoxicity and even second malignancy.^47^ Therefore, it is of great significance to find novel therapeutic agents to replace dexrazoxane. In the present study, we found that cardiac *miR*‐*495*‐*3p* expression was significantly decreased in Dox‐treated hearts, and that the *miR*‐*495*‐*3p* agomir could prevent oxidative stress, cell apoptosis, cardiac mass loss, fibrosis and cardiac dysfunction upon Dox stimulation. In addition, we demonstrated that *miR*‐*495*‐*3p* directly bound to the 3′‐UTR of PTEN, downregulated PTEN expression and subsequently activated AKT pathway, and that PTEN overexpression or AKT inhibition completely abolished the cardioprotective effects of the *miR*‐*495*‐*3p* agomir (Figure 8). Overall, our study for the first time identify *miR*‐*495*‐*3p* as an endogenous protectant against Dox‐induced cardiotoxicity in vivo and in vitro.

Oxidative stress is a key feature of Dox‐induced cardiotoxicity, and contributes to cell loss and cardiac dysfunction. It has been reported that Dox is specifically and abundantly accumulated in the heart, and exhibits high affinity to cardiolipin. The formation of Dox‐cardiolipin complex anchors Dox to the inner membrane of mitochondria that disturbs normal electron transport chain and promotes ROS generation in the heart.^48^ In addition, the redox‐cycling of Dox generates massive semiquinone free radicals and exacerbates oxidative stress.^49^ Iron is also essential for Dox‐induced ROS generation; however, the free iron level within most cells is lower than the threshold to couple with Dox to the extent necessary to cause cardiotoxicity. Emerging studies have identified that Dox interferes the proteins that sequester and bind intracellular iron, thereby increasing the accumulation of iron inside the mitochondria and promoting ROS amplification through a Fenton reaction.^7^ Excessive free radicals induce oxidative damage to biomacromolecules and destroy cellular physiology. Moreover, the less active antioxidant defence and negligible regenerative capability of the heart further exacerbate Dox‐induced cardiotoxicity.^8^ Accordingly, previous studies by us and the others have found that suppressing oxidative stress is sufficient to prevent Dox‐induced cardiac injury and dysfunction.^9^, ^46^

miRNAs belong to the family of small noncosding RNAs and participate in various biological processes through negatively regulating gene expression.^50^, ^51^ Dysregulated miRNAs have been identified in the pathogenesis of Dox‐induced cardiotoxicity, and even can be regarded as serum biomarkers for cardiac injury in Dox‐treated patients.^52^, ^53^ We previously demonstrated that *miR*‐*22* was elevated in murine hearts with chronic Dox injection, and that *miR*‐*22* inhibition reduced Dox‐induced oxidative stress, cardiomyocyte apoptosis and cardiac dysfunction in vivo and in vitro.^9^ *miR*‐*495*‐*3p* expression is altered in various human tumours, and exhibits high potency to inhibit tumour growth and chemoresistance.^22^, ^23^ Herein, we determined that treatment with the *miR*‐*495*‐*3p* agomir could alleviate Dox‐induced cardiotoxicity in vivo and in vitro. Based on these findings, we reasonably define *miR*‐*495*‐*3p* as an effective and safe target to treat Dox‐induced cardiotoxicity. Of course, there are some limitations of the present study. First, the primary cellular source of *miR*‐*495*‐*3p* and its targeted cells have not been completely determined in this study. Second, the exact mechanisms mediating the cardioprotection of *miR*‐*495*‐*3p* except AKT pathway remains unknown. Third, whether the *miR*‐*495*‐*3p* agomir can sensitize human tumour cells to Dox chemotherapy needs further investigation.

In summary, we demonstrate that *miR*‐*495*‐*3p* diminishes Dox‐induced cardiotoxicity through activating AKT pathway, and targeting *miR*‐*495*‐*3p* may provide novel cardioprotective approaches for cancer patients receiving anthracycline chemotherapy.

## CONFLICT OF INTEREST

The authors declare no conflict of interest.

## AUTHOR CONTRIBUTIONS

**Jun Meng:** Conceptualization (equal);– review and editing data curation (equal); formal analysis (equal); investigation (equal); methodology (equal); software (equal); supervision (equal); validation (equal); visualization (equal); writing (equal). **Can Xu:** Conceptualization (equal); data curation (equal); formal analysis (equal); funding acquisition (equal); investigation (equal); methodology (equal); software (equal); supervision (equal); validation (equal); writing – original draft (equal); writing – review and editing (equal).

## Data Availability

The research data are not shared.
